# Unravelling excitation/inhibition imbalance in early and familial Alzheimer's Disease

**DOI:** 10.1002/alz.084939

**Published:** 2025-01-09

**Authors:** Anne M van Nifterick

**Affiliations:** ^1^ Amsterdam UMC location Vrije Universiteit Amsterdam, Department of Clinical Neurophysiology and MEG Center, Neurology, De Boelelaan 1117, Amsterdam Netherlands

## Abstract

**Background:**

An early disruption in excitation‐inhibition (E‐I) balance is believed to underlie abnormal local and global network dynamics in Alzheimer’s disease (AD). While prior studies proposed that changes in E‐I balance may underlie the established slowing of oscillatory activity and changes in functional connectivity in symptomatic AD, the specific alterations occurring in the presymptomatic stage of AD remain poorly understood. This study aims to identify local and global spectral power and functional connectivity changes in individuals with familial AD before the onset of clinical symptoms.

**Method:**

Resting‐state magnetoencephalography (MEG) recordings were conducted in a unique cohort of cognitively unimpaired individuals carrying autosomal dominant mutations in the APP and PSEN1 genes (n = 11), leading to early‐onset AD. Each mutation carrier was demographically matched with three cognitively healthy subjects (HCs) without mutations and with available MEG. We compared source‐level whole‐brain average and regional spectral power and volume‐conduction corrected functional connectivity measures across different frequency bands. Hub vulnerability was assessed using the hub disruption index. Significant MEG measures were correlated with age, estimated years before symptom onset (EYBSO), and cognitive performance.

**Result:**

Cognitively normal mutation carriers exhibited signatures of oscillatory slowing, characterized by a widespread higher relative power in the theta band (4‐8 Hz) and a lower parieto‐temporal‐occipital peak frequency compared to HCs. Additionally, they presented frequency‐ and degree‐dependent functional connectivity changes: higher phase‐based functional connectivity in the theta band in initial low‐degree regions (as determined by weighted degree in HCs), but lower global amplitude‐based functional connectivity in the alpha (8‐13 Hz) and beta (13‐30 Hz) bands compared to HCs, most strongly in initial high‐degree regions. No correlations between MEG measures and age, EYBSO, or cognitive indices were found after correction for multiple comparisons.

**Conclusion:**

Neurophysiological changes occur before the onset of cognitive symptoms in individuals carrying mutations in the APP or PSEN1 genes. These changes include oscillatory slowing and frequency‐ and degree‐dependent functional connectivity alterations, mirroring alterations observed in clinical stages of AD and suggesting an early E‐I imbalance. These findings contribute to a comprehensive understanding of presymptomatic pathophysiological alterations in AD.

